# Bioinformatic Evaluation of *KLF13* Genetic Variant: Implications for Neurodevelopmental and Psychiatric Symptoms

**DOI:** 10.3390/genes15081056

**Published:** 2024-08-11

**Authors:** Mirella Vinci, Donatella Greco, Simone Treccarichi, Valeria Chiavetta, Maria Grazia Figura, Antonino Musumeci, Vittoria Greco, Concetta Federico, Francesco Calì, Salvatore Saccone

**Affiliations:** 1 Oasi Research Institute-IRCCS, 94018 Troina, Italy; mvinci@oasi.en.it (M.V.); dgreco@oasi.en.it (D.G.); streccarichi@oasi.en.it (S.T.); vchiavetta@oasi.en.it (V.C.); mfigura@oasi.en.it (M.G.F.); amusumeci@oasi.en.it (A.M.); 2 Department of Biomedical Science, University of Messina, 98122 Messina, Italy; vgreco@oasi.en.it; 3 Department of Biological, Geological and Environmental Sciences, University of Catania, Via Androne 81, 95124 Catania, Italy; concetta.federico@unict.it (C.F.); salvatore.saccone@unict.it (S.S.)

**Keywords:** Krüppel-like factor, axonal growth, transcription factors, next-generation sequencing, missense mutation, autosomal dominant inheritance

## Abstract

The Krüppel-like factor (KLF) family represents a group of transcription factors (TFs) performing different biological processes that are crucial for proper neuronal function, including neuronal development, synaptic plasticity, and neuronal survival. As reported, genetic variants within the KLF family have been associated with a wide spectrum of neurodevelopmental and psychiatric symptoms. In a patient exhibiting attention deficit hyperactivity disorder (ADHD) combined with both neurodevelopmental and psychiatric symptoms, whole-exome sequencing (WES) analysis revealed a de novo heterozygous variant within the Krüppel-like factor 13 (*KLF13*) gene, which belongs to the KLF family and regulates axonal growth, development, and regeneration in mice. Moreover, in silico analyses pertaining to the likely pathogenic significance of the variant and the impact of the mutation on the KLF13 protein structure suggested a potential deleterious effect. In fact, the variant was localized in correspondence to the starting residue of the N-terminal domain of *KLF13*, essential for protein–protein interactions, DNA binding, and transcriptional activation or repression. This study aims to highlight the potential involvement of the *KLF13* gene in neurodevelopmental and psychiatric disorders. Nevertheless, we cannot rule out that excluded variants, those undetectable by WES, or the polygenic risk may have contributed to the patient’s phenotype given ADHD’s high polygenic risk. However, further functional studies are required to validate its potential contribution to these disorders.

## 1. Introduction

The Krüppel-like factor (KLF) family of transcription factors plays a role in controlling various biological functions, such as cell growth, differentiation, development, and survival, as well as in embryonic development, tissue differentiation, metabolism, and reactions to environmental stress [1,2]. The typical trait of the KLF family is the presence of three Krüppel-like zinc finger functional domains. These zinc fingers have an affinity for binding to CACCC elements and GC-rich regions of DNA, enabling the mediation of transcriptional activation and/or repression [2,3].

KLFs have emerged as key regulators in the pathogenesis of various neurological diseases. Their involvement spans a wide range of processes that are crucial for proper neuronal function, including neuronal development, synaptic plasticity, and neuronal survival [4,5,6]. The dysregulation of KLFs has been implicated in several neurological disorders, including Alzheimer’s disease (AD), Parkinson’s disease (PD), epilepsy, and intellectual disabilities (ID). For instance, altered expression levels of KLFs have been associated with the aberrant synaptic transmission and neuroinflammation observed in AD [7,8]. Similarly, in PD, dysregulated KLFs have been linked to neuronal apoptosis and impaired dopamine signaling pathways [9,10,11]. Moreover, mutations in genes encoding KLF proteins have been identified in individuals with ID, suggesting the critical role of KLFs in neurodevelopmental processes [12]. In fact, KLF transcription factors have been found to be engaged in late-phase neuronal maturation in the developing dentate gyrus and during adult hippocampal neurogenesis [13]. For instance, as outlined in diverse studies, *KLF7* was recently associated with developmental delay/ID and neuromuscular and psychiatric symptoms [12,14,15]. Additionally, it was associated with learning disorders and autistic behaviors [8,16]. Overall, understanding the intricate involvement of KLFs in neurological diseases holds significant promise for the development of novel therapeutic strategies aimed at modulating KLF activity to mitigate disease progression and improve patient outcomes [10,17,18].

Among the wide array of KLF genes, *KLF13* stands as an important transcription factor containing three classical zinc finger DNA-binding domains consisting of a zinc atom tetrahedrally coordinated by two cysteines and two histidines (C2H2 motif) [19,20,21,22]. According to diverse expression databases and studies, *KLF13* is ubiquitously expressed in various tissue types, including the hippocampus and the central nervous system [23,24]. As recently documented, it was found to be a booster for myelin gene expression in oligodendrocytes in the central nervous system [25]. Moreover, KLF13 has been identified as a crucial regulator of heart development and is involved in various cardiac functions [26,27,28,29]. Additionally, it seems to play a significant role as a cross-talking regulator in cancer across different tissue types, including oral cells, the pancreas, the prostate, and the stomach [30,31,32,33,34].

Within this context, the objective of the current manuscript is to highlight the potential implications of a genetic variant found within *KLF13* in a patient showing neurodevelopmental and psychiatric symptoms encompassing attention deficit and hyperactivity disorder (ADHD).

## 2. Materials and Methods

### 2.1. Library Preparation and Next-Generation Sequencing (NGS)

Genomic DNA was obtained from the patient’s and their parents’ peripheral blood leukocytes, as previously described [35]. Library preparation (TRIOS) and exome enrichment were performed employing the Agilent SureSelect V7 Kit (Santa Clara, CA, USA), following the manufacturer’s instructions. The sequencing run was carried out on an Illumina HiSeq 3000 instrument (San Diego, CA, USA). This specific method allowed the achievement of 97% of regions covered at least 20× The identified variants were filtered based on (i) a recessive/de novo/X-linked pattern of inheritance; (ii) allele frequencies (mean average frequency, MAF) < 1%, using, as a reference, the following genomic datasets: 1000 Genomes, ESP6500, ExAC, GnomAD. Notably, the reference genome was HG38. The confirmation of the de novo event was achieved through conventional Sanger sequencing using the BigDyeTM Terminator v1.1 Cycle Sequencing Kit (Life Technologies, Carlsbad, CA, USA) with the SeqStudio Genetic Analyzer instrument (Thermo Fisher Scientific, Waltham, MA, USA). The sequences of the primers employed for the trial were for. 5′-TGACGACTCGCAGCAAGAG-3′, rev. 5′-CTGGTTGAGGTCCGCTAGGA-3′. In alignment with a previous protocol [36], DNA fingerprint analysis was carried out to confirm maternity and paternity for both the patient and parents.

### 2.2. Data Analysis

The variant was searched on the Human Gene Mutation Database (HGMD Professional 2023). Furthermore, it was filtered employing VarAft (2.17-2) [37]. The described variant was classified in alignment with the “American College of Medical Genetics” (ACMG) guidelines [38] through VarSome, according to previous research [39]. The criteria adopted for variant classification are listed in Table 1.

The prediction of the inheritance pattern of the *KLF13* gene was performed by DOMINO (https://domino.iob.ch/) (accessed on 6 June 2024), as previously described, assigning a probability score ranging from 0 (recessive) to 1 (autosomal dominant) [40]. The *KLF13* gene expression patterns were investigated using the GTEx (https://www.gtexportal.org/home/) (accessed on 6 June 2024) and Human Protein Atlas (HPA) (https://www.proteinatlas.org/) (accessed on 6 June 2024) databases. PhastCons100way and PhyloP100way scores (from VarSome analysis) were used to analyze the conservation tendency of the specific mutation region. Gene ontology (GO) terms related to the protein and functional domain annotations were obtained via the QuickGO database (https://www.ebi.ac.uk/QuickGO/) (accessed on 6 June 2024), in addition to the Uniprot (https://www.uniprot.org/) (accessed on 6 June 2024), InterPro (https://www.ebi.ac.uk/interpro/) (accessed on 6 June 2024), and Prosite (https://prosite.expasy.org/) (accessed on 6 June 2024) databases. Protein–protein interactions based on experimental studies were investigated on the STRING and IntAct (https://www.ebi.ac.uk/intact/home) (accessed on 6 June 2024) databases. Protein structure predictions were generated employing the UCSF ChimeraX software version 1.7. The structure analysis was conducted based on the AlphaFold algorithm, generating five models and selecting the “best model”, as previously described [41]. Protein stability changes were predicted using the in silico tools DUET (https://biosig.lab.uq.edu.au/duet/) (accessed on 6 June 2024) and PONDR (http://pondr.com/) (accessed on 6 June 2024). The heatmap and dendrogram for the 18 KLF proteins, based on the percentage of sequence identity, were generated using RStudio version 3.6.3 with the following packages: ggplot2, pheatmap, ggdendro, and dplyr. Additionally, the line plots for PONDR analysis were created using the ggplot2 package.

## 3. Results

### 3.1. Clinical Report

We describe a 10-year-old boy diagnosed with ADHD—combined presentation and a specific learning disorder (SLD) according to the DSM-5 criteria. He was the firstborn of non-consanguineous parents, from a normal pregnancy that required an urgent cesarean section due to complications during labor, although no perinatal distress was reported. His birth weight was 2970 g, and he had mild jaundice. He was breastfed for three months and weaned without complications. His psychomotor development was normal; he started walking at 14 months, talking at 18 months, and speaking in full sentences by 24 months.

He first presented to a child neuropsychiatrist at age 5 due to behavioral problems, such as irritability with destructive rages, hyperactivity, and impulsivity with a lack of awareness of danger. He was diagnosed with oppositional defiant disorder. At age 7, he was observed to exhibit sudden and unexplained behavioral worsening during his second year of primary school, including extreme tantrums, violent aggressive outbursts, and cognitive rigidity. After administering tests and multi-evaluation checklists (WISC IV, CPRS-R, CBCL, CTRS-R), he was diagnosed with combined-type, moderate ADHD. His treatment plan included 10 mg of immediate-release methylphenidate twice a day, resulting in a remarkable improvement. Upon discharge, the methylphenidate dosage was titrated up to 30 mg per day (modified release).

The phenotype was characterized by excess weight with a BMI of 23.1 kg/m^2^ (95th percentile), myopic astigmatism, a rounded face, small eyes, a wide nose with a bulbous tip, small ears with hypoplastic lobes, fleshy lips with an everted lower lip, short distal phalanges, and ligament hyperlaxity. Cardiac and thoracic examinations were normal. Other systemic examinations were unremarkable, and the EEG was normal

### 3.2. Next-Generation Sequencing (NGS)

**Figure 1:**
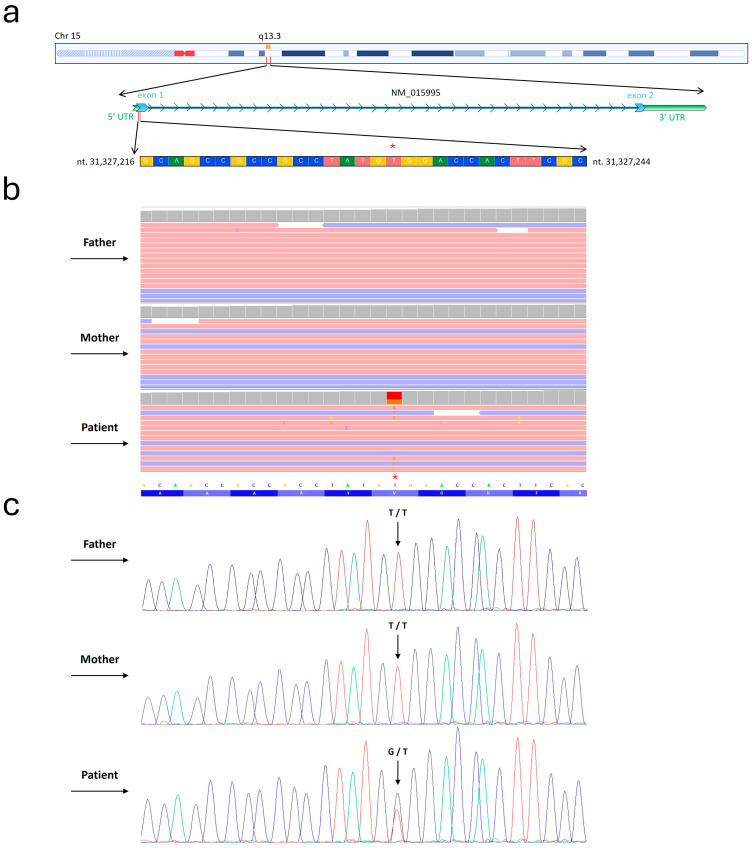
Detection of c.20T>G within *KLF13* gene. (**a**) Depiction of the nucleotide sequence corresponding to the region where the mutation was identified within the *KLF13* gene. Furthermore, the chromosomal localization of this gene is illustrated. Figure was modified from the UCSC genome database. The asterisk indicates the precise variant site. (**b**) Whole-exome sequencing (WES) results are presented using the Integrative Genomics Viewer (IGV) visualization tool. As shown in the picture, WES was carried out for the examined patient and both healthy parents. (**c**) Conventional Sanger sequencing was performed to highlight the c.20T>G variant identified by WES. In the electropherograms, the black, blue, green, and red profiles indicate nucleotides G, C, A, and T.

WES Trio analysis unveiled a de novo heterozygous variant (c.20T>G) within the *KLF13* gene (NM_015995) (Figure 1).

NGS analysis did not identify genetic variants in genes known to be associated with the patient’s symptoms. The variant was confirmed by conventional Sanger sequencing. DOMINO analysis indicated that the *KLF13* gene displayed an autosomal dominance inheritance pattern, with a probability score of 0.96.

### 3.3. Protein Structure Prediction and Pathway Analysis

**Figure 2:**
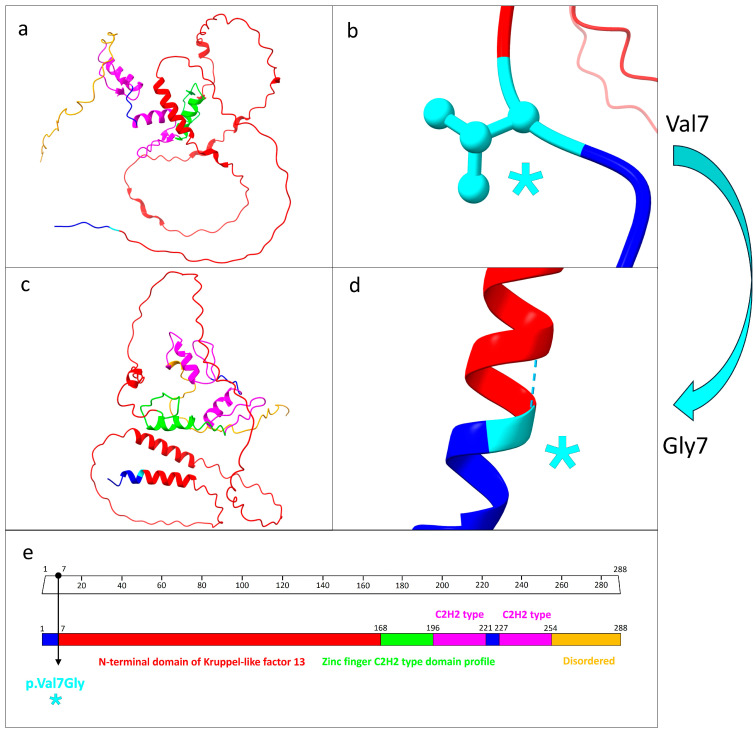
Structure prediction analysis and functional domains related to KLF13 protein. (**a**) Protein structure prediction related to the wild-type KLF13. Each functional domain is marked by different colors. (**b**) Focus on the wild-type valine residue at position 7, which did not engage in hydrogen bonds with other amino acids. (**c**) Mutated KLF13 protein. As predicted, the different structural protein folding as result of the mutation is evident. (**d**) Close-up of the mutated residue as a result of the missense mutation p.Val7Gly. (**e**) Domain organization patterns related to the KLF13 protein. The specific mutation site is indicated by the black arrow. The light blue asterisk in (**b**,**d**,**e**) indicates the precise position of the missense mutation. (**a**–**d**) were generated by UCSF ChimeraX software, while (**e**) was modified from Uniprot database.

This novel variant was a missense mutation (p.Val7Gly) localized at position 7, corresponding to the first amino acid of the N-terminal domain of KLF13 (from aa 7 to aa 168) (Figure 2).

The PhyloP100way and the PhastCons100way scores related to the conservation rate among 100 vertebrate species of the specific mutated site at position 7 were 5.905 and 1, respectively. This indicates the high conservation of valine 7 among various species.

In comparison to the various KLF proteins, the protein identity percentage of KLF13 among all 18 KLF proteins ranges from 25.68% for KLF18 to 53.77% for KLF6 (Figure S1a). Notably, KLF13 exhibits the highest sequence similarity with KLF6 (53.77%), KLF9 (48.10%), and KLF16 (51.11%), as shown in the dendrogram based on the sequence similarity (Figure S1b).

The protein structure prediction analysis conducted using UCSF ChimeraX revealed a significant structural alteration between the wild-type and mutated forms of KLF13. Specifically, the number of interprotein hydrogen bonds increased from 111 in the wild type to 123 in the mutated form. Furthermore, while the wild-type valine residue at position 7 lacked hydrogen bonds, the mutated glycine residue at the same position formed a hydrogen bond with alanine at position 11 (Figure 2).

**Figure 3:**
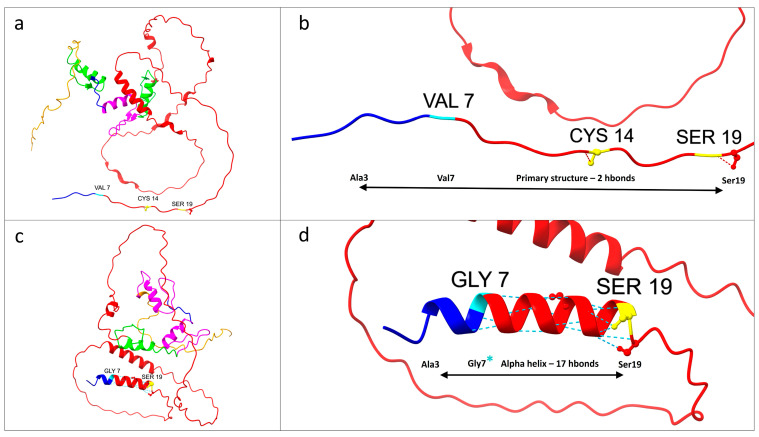
Structure prediction analysis of the KLF13 protein, focusing on the amino acid residues from positions 3 to 19, revealed significant structural variation from the primary structure to an α helix. Notably, the N-terminal domain of Krüppel-like factor 13 (from residues 7 to 168) begins at the specific mutation site at amino acid 7. The colors used are consistent with the domain organization patterns shown in Figure 2. (**a**) Wild-type KLF13 protein. (**b**) Close-up of the wild-type protein segment from alanine 3 to serine 19. (**c**) Mutated KLF13 protein, with visibly different predicted protein folding compared to the wild type. (**d**) Close-up of the segment from alanine 3 to serine 19, highlighting the mutated glycine 7 (marked with an asterisk). The missense mutation is predicted to result in the formation of an α helix containing 17 hydrogen bonds. (**a**–**d**) were generated by UCSF ChimeraX software and subsequently modified.

As is clearly depicted in Figure 2 and Figure 3, as predicted, the mutation caused the generation of an α helix from alanine at position 3 to serine at position 19.

The predicted new α helix, consisting of 16 amino acids, resulted in a higher number of hydrogen bonds (17 hydrogen bonds) in comparison to the wild-type primary structure (two hydrogen bonds).

**Figure 4:**
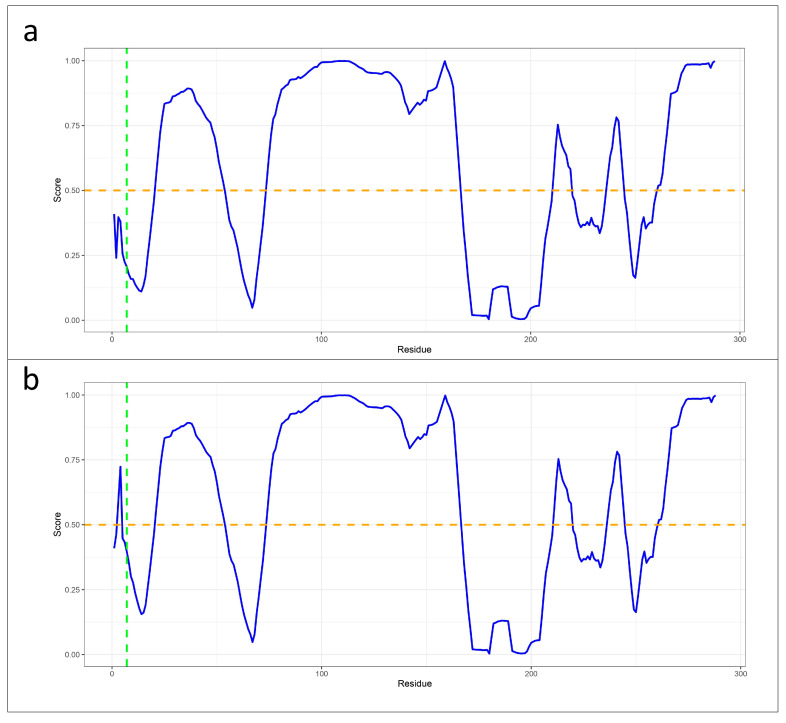
Line plots generated with PONDR tool with VLXT score to assess the impact of the mutation on protein stability and flexibility. (**a**) The VLXT score (blue line) from PONDR analysis for the wild-type KLF13 protein indicates a high rate of structural order at the specific site (green line indicating valine at position 7), with scores lower than 0.5 (orange line). (**b**) The VLXT score from PONDR analysis for the mutated KLF13 protein shows a higher rate of disorder as a result of the mutation. This is evidenced by the values of the residues before the mutation site being higher than 0.5 (orange line).

The analysis conducted using MuPRO to assess the impact of the mutation on the protein structure revealed a decrease in protein stability. Specifically, the mutation was assigned a delta delta G score of −2.175, indicating a significant destabilizing effect on the protein. In the protein stability analysis performed with the DUET tool, a significant structural variation was observed, resulting in the destabilizing effect of the mutation. This was reflected in the values of the mCSM, SDM, and DUET scores, which indicated a predicted stability change of −0.437 kcal mol^−1^, −0.51 kcal mol^−1^, and −0.239 kcal mol^−1^ at the mutated point, respectively. In addition, MutPred2 indicated that the amino acid change can lead to the gain of protein intrinsic disorder, as well to an alteration in the metal-binding activity. Furthermore, the protein disorder analysis conducted using PONDR revealed a structural alteration in KLF13 due to the genetic variant, resulting in an elevated level of disorder in residues three and four compared to the wild-type protein (Figure 4).

According to the STRING and IntAct databases, supported by various experimental studies, the KLF13 protein has been found to correlate with the CHCHD10, KAT2B, and MMP28 proteins.

## 4. Discussion

In the current manuscript, we present a clinical case exhibiting neurodevelopmental and psychiatric symptoms encompassing ADHD. WES Trio analysis identified a heterozygous de novo variant within *KLF13*. This gene belongs to the KLFs, a gene family widely associated with a broad spectrum of neurological conditions [14,42]. In the WES analysis performed, variants with an allele frequency greater than 1%, as well as those deemed benign according to the ACMG criteria, were excluded. The variant described in the current manuscript was deemed likely pathogenic according to the ACMG criteria listed in Table 1. Furthermore, the WES analysis revealed no variants in the candidate genes for ADHD, consistent with the filters applied. Nonetheless, we cannot rule out the possibility that the excluded variants, along with other genetic factors undetectable by WES—such as mutations in non-coding regions or the polygenic risk involving inter-allelic complementation—may have contributed to the patient’s phenotype, given the high polygenic risk associated with ADHD. We hypothesize a potential correlation between *KLF13* and the patient’s symptoms due to its ontology and its role as a transcriptional repressor in various pathways, including JAK/STAT signaling [43].

Additionally, the phenotype examined in the current study was characterized by excess weight, with a BMI of 23.1 kg/m^2^ (95th percentile). The association found in the literature between *KLF13* methylation and orexigenic features, as well as digestive physiology and obesity [23,44,45], may be attributed to inflammatory conditions resulting from altered KLF13 activity. Variations in KLF13 can disrupt the chemokine systems, which are closely linked to energy metabolism, insulin resistance, and obesity. Additionally, KLF13 has been reported as a pro-adipogenic transcription factor and a key regulator of adipocyte differentiation [46,47].

The identified variant was not described in either the Exome Aggregation Consortium (ExAC) or the 1000 Genomes Project (1000G) databases. Furthermore, the allele frequency of the variant was not found in the Genome Aggregation Database (GnomAD). According to ACMG criterion BP4, described in Table 1, the in silico analysis did not provide sufficient evidence to classify the variant as likely pathogenic. However, based on criteria PS2, PM2, and PP4, the variant was classified as likely pathogenic (Table 1). According to the DOMINO analysis, the KLF13 gene displays an autosomal inheritance pattern. Additionally, both the PhastCons100way and PhyloP100way analyses indicated that the specific mutated site is a well-conserved region among 100 vertebrate species, suggesting its likely functional relevance in the KLF13 gene across different species.

The described missense variation localized at the starting amino acid of the N-terminal domain of KLF13 (from aa 7 to aa 168). In transcription factors like KLF13, the N-terminal domain is crucial for various functional aspects, such as protein–protein interactions, DNA binding, transcriptional activation or repression, and subcellular localization [48,49,50]. This domain plays a critical role in regulating the protein’s activity and its ability to modulate gene expression. It is worth noting that the N-terminal domain exhibits significant variability among the KLF transcription factors, highlighting their functional diversity [49]. This variability in the structural motifs outside the DNA-binding domain is associated with the functional diversity observed within the KLF family [51]. In fact, according to the InterPro database, each KLF also has a unique N-terminal activation/repression domain that confers specificity and allows it to bind specifically to a certain partner, leading to distinct activity in vivo. It is worth noting that the percentage of protein identity between KLF13 and other KLF proteins ranges from 25.68% for KLF18 to 53.77% for KLF6 (Figure S1). Furthermore, among the 18 KLF proteins, only KLF1, KLF6, and KLF11 have MIM phenotype numbers, associating them with dyserythropoietic anemia (613673), prostate (176807) and gastric (613659) cancers, and maturity-onset diabetes of the young, type VII (610508), respectively. This wide array of diseases associated with these proteins underscores the intricate functional diversity of the KLF domain among the KLF genes.

Additionally, the structural prediction analysis revealed notable disparities between the wild-type and mutated proteins. Specifically, employing UCSF ChimeraX, it was observed that the mutated protein displayed an increase in the total number of hydrogen bonds by 12 compared to the wild type. Specifically, as evidenced by the structural prediction analysis, the mutation altered the protein folding, causing the generation of an α helix from the residues alanine 3 to serine 19 (Figure 3, Table S1). Compared to the wild-type primary structure predicted by AlphaFold, the new structure exhibited the rearrangement of the hydrogen bond patterns in the abovementioned portion, resulting in an increase from two hydrogen bonds in the wild type to 17 in the mutated protein. This predicted variation encompasses the beginning of the N-terminal domain of KLF13, suggesting a potential alteration in its DNA-binding functional activity. Furthermore, both the DUET and MuPRO algorithms indicated a destabilizing effect of the identified variant on the protein structure, resulting in decreased protein stability as a consequence of the genetic alteration. Moreover, MutPred2 suggested a plausible impact of the mutation on the gain of intrinsic protein disorder and the potential alteration of metal-binding sites. The PONDR analysis revealed an increase in protein disorder for the residues before the mutated amino acid at position 7, in comparison to the wild-type protein (Figure 4).

As concerns the gene ontology annotations related to the transcription factor KLF13, it enables RNA polymerase II cis-regulatory region sequence-specific DNA binding (GO:0000978), DNA-binding transcription factor activity, RNA polymerase II-specific (GO:0000981 and GO:0001228), DNA binding (GO:0003677), protein binding (GO:0005515), metal ion binding (GO:0046872), and sequence-specific double-stranded DNA binding (GO:1990837). Furthermore, as annotated, it is engaged in the regulation of transcription by RNA polymerase II (GO:0006357) and transcription by RNA polymerase II (GO:0006366), the negative regulation of cell population proliferation (GO:0008285), the negative regulation of erythrocyte differentiation (GO:0045647), and the positive regulation of transcription by RNA polymerase I (GO:0045944). Additionally, as annotated, KLF13 is localized in the nucleus (GO:0005634) and ultimately it is part of the chromatin (GO:0000785). The pathway analysis revealed that genes regulated by *KLF13* are implicated in various processes, including cell cycle regulation, cell survival, and cytoarchitecture regulation, among others [24]. As is well known, dendritic anomalies in the hippocampal tissue can lead to neurodevelopmental disorders [52,53,54].

As documented, KLF13 acts as a transcriptional repressor, modulating several signaling pathways that are crucial in the central nervous system (CNS), including the JAK/STAT pathway [43,55,56,57]. This pathway serves as the canonical mediator of growth hormone (GH) signaling, known for its increasingly recognized neurotrophic effects associated with synaptic plasticity and neuronal stem cells [58,59]. Alterations in this pathway have been linked to imbalances in dopaminergic receptors [60,61,62], which are closely involved in ADHD as well in neurodevelopmental and psychiatric disorders [63,64,65]. It has been suggested that *KLF13* could play a role in maintaining the homeostatic state of this pathway [43,66,67]. As previously reported, *KLF13* displays high expression patterns in diverse brain tissue types (GTEx; HPA). Within this context, it was recently outlined that *KLF13* boosts the expression of the myelin gene in oligodendrocytes in the central nervous system [25]. Additionally, the expression of *Klf13* increases in the hippocampus during postnatal development in mice, similarly to its paralog *Klf9*, supporting their role in promoting and maintaining neuronal differentiation [24,68]. Furthermore, *KLF13* has been found to play a crucial role in hippocampal cell function, proliferation, survival, and regeneration. It predominantly acts as a transcriptional repressor in hippocampal neurons and can also regulate other KLF genes [24]. Both KLF9 and KLF13, in fact, primarily function as transcriptional repressors by associating with chromatin in the proximal promoters of target genes, inhibiting axon growth by repressing key components of the cAMP signaling pathway.

In population-based studies, clinical assessments for ADHD are often difficult to predict due to the diagnostic heterogeneity and varying comorbidities [69,70]. As outlined, ADHD has a high genetic contribution, with heritability estimated at 74% [71,72,73]. The regulation of gene expression is crucial in neurological processes, impacting chromatin accessibility through histone modifications, DNA methylation, and chromatin remodeling [74,75,76]. Considering *KLF13*’s role in chromatin modification and remodeling [77,78,79], we suppose that variations in its function might lead to epigenetic changes, such as DNA methylation, which contribute to ADHD [80,81]. Despite the high polygenic risk and complex genetic architecture of ADHD, we hypothesize that *KLF13* might have contributed to the patient’s intriguing phenotype, either individually or in interaction with other proteins.

According to the STRING and IntAct databases, supported by various experimental studies, the KLF13 protein has been found to correlate with the CHCHD10, KAT2B, and MMP28 proteins [77,82,83,84]. Notably, according to the OMIM database, *CHCHD10* is associated with frontotemporal dementia and/or amyotrophic lateral sclerosis 2 (615911), which encompasses a wide range of neurodegenerative conditions [7,85]. Furthermore, KAT2A plays a crucial role in hippocampal synaptic plasticity and long-term memory consolidation, as evidenced by studies showing defects in these processes in mice lacking Kat2a [86,87]. Additionally, MMP28 has been linked to learning and memory processes [88,89]. Based on these documented interactions, we hypothesize that alterations in the KLF13 protein may potentially impact its interaction with the abovementioned genes, although further studies are required to elucidate this.

Further investigations and functional studies are imperative to confirm the role of this gene in neurological and psychiatric symptoms.

## 5. Conclusions

The present manuscript documents a clinical case involving ADHD combined with neurologic and psychiatric conditions. WES Trio analysis identified a heterozygous de novo variant within KLF13, a gene belonging to the KLF family, known as transcription factors and involved in various molecular functions, such as DNA, protein, and metal ion binding. In this manuscript, we explore the potential association between the KLF13 gene and the patient’s phenotype based on in silico predictions and variant classification according to the ACMG criteria. However, further validation through functional studies is necessary to confirm these findings.

## Figures and Tables

**Table 1:** Criteria used for the classification of the observed nucleotide variation.

Criteria for Classification of Variants	Category Code	Description
Strong	PS2	De novo (both maternity and paternity confirmed) in a patient with the disease and no family history.
Moderate	PM2	Absent from controls (or at extremely low frequency if recessive) in Exome Sequencing Project, 1000 Genomes Project, or Exome Aggregation Consortium.
Supporting	PP4	Patient’s phenotype or family history is highly specific for a disease with a single genetic etiology.
Benign supporting	BP4	Multiple lines of computational evidence suggest no impact on gene or gene product (conservation, evolutionary, splicing impact)
ACMG variant classification		Likely pathogenic

## Data Availability

The data presented in this study are given in the main text and in the Supplementary File.

## Supplementary Materials

The following supporting information can be downloaded at: https://www.mdpi.com/article/10.3390/genes15081056/s1, Figure S1: Identity levels of 18 KLF proteins; Table S1: Variations in the hydrogen bond patterns.

## References

[1] McConnell B.B., Yang V.W. (2010). Mammalian Krüppel-Like Factors in Health and Diseases. Physiol. Rev..

[2] Presnell J.S., Schnitzler C.E., Browne W.E. (2015). KLF/SP Transcription Factor Family Evolution: Expansion, Diversification, and Innovation in Eukaryotes. Genome Biol. Evol..

[3] Pearson R., Fleetwood J., Eaton S., Crossley M., Bao S. (2008). Krüppel-like Transcription Factors: A Functional Family. Int. J. Biochem. Cell Biol..

[4] Toda T., Gage F.H. (2018). Review: Adult Neurogenesis Contributes to Hippocampal Plasticity. Cell Tissue Res..

[5] Chen Y., Chen J., Chen Y., Li Y. (2022). MiR-146a/KLF4 Axis in Epileptic Mice: A Novel Regulator of Synaptic Plasticity Involving STAT3 Signaling. Brain Res..

[6] Kennedy C.L.M., Price E.M., Mifsud K.R., Salatino S., Sharma E., Engledow S., Broxholme J., Goss H.M., Reul J.M.H.M. (2023). Genomic Regulation of Krüppel-like-Factor Family Members by Corticosteroid Receptors in the Rat Brain. Neurobiol. Stress.

[7] Yi R., Chen B., Zhao J., Zhan X., Zhang L., Liu X., Dong Q. (2014). Krüppel-like Factor 8 Ameliorates Alzheimer’s Disease by Activating β-Catenin. J. Mol. Neurosci..

[8] Tian H., Jiao Y., Guo M., Wang Y., Wang R., Wang C., Chen X., Tian W. (2022). Krüppel-like Factor 7 Deficiency Causes Autistic-like Behavior in Mice via Regulating Clock Gene. Cell Biosci..

[9] Chen J., Wang X., Yi X., Wang Y., Liu Q., Ge R. (2013). Induction of KLF4 Contributes to the Neurotoxicity of MPP + in M17 Cells: A New Implication in Parkinson’s Disease. J. Mol. Neurosci..

[10] Zamanian M.Y., Golmohammadi M., Amin R.S., Bustani G.S., Romero-Parra R.M., Zabibah R.S., Oz T., Jalil A.T., Soltani A., Kujawska M. (2024). Therapeutic Targeting of Krüppel-Like Factor 4 and Its Pharmacological Potential in Parkinson’s Disease: A Comprehensive Review. Mol. Neurobiol..

[11] El-Deeb A.M., Mohamed A.F., EL-Yamany M.F., El-Tanbouly D.M. (2023). Novel Trajectories of the NK1R Antagonist Aprepitant in Rotenone-Induced Parkinsonism-like Symptoms in Rats: Involvement of ERK5/KLF4/P62/Nrf2 Signaling Axis. Chem. Biol. Interact..

[12] Hong W., Gong P., Pan X., Liu Y., Qi G., Qi C., Qin S. (2023). Krüppel-like Factor 7 Deficiency Disrupts Corpus Callosum Development and Neuronal Migration in the Developing Mouse Cerebral Cortex. Brain Pathol..

[13] Scobie K.N., Hall B.J., Wilke S.A., Klemenhagen K.C., Fujii-Kuriyama Y., Ghosh A., Hen R., Sahay A. (2009). Krüppel-Like Factor 9 Is Necessary for Late-Phase Neuronal Maturation in the Developing Dentate Gyrus and during Adult Hippocampal Neurogenesis. J. Neurosci..

[14] Powis Z., Petrik I., Cohen J.S., Escolar D., Burton J., van Ravenswaaij-Arts C.M.A., Sival D.A., Stegmann A.P.A., Kleefstra T., Pfundt R. (2018). De Novo Variants in *KLF7* Are a Potential Novel Cause of Developmental Delay/Intellectual Disability, Neuromuscular and Psychiatric Symptoms. Clin. Genet..

[15] Mao Y., Chen Y., Zhang Z. (2023). Molecular Function of Krüppel-like Factor 7 in Biology. Acta Biochim. Biophys. Sin..

[16] Roberts J.L., Hovanes K., Dasouki M., Manzardo A.M., Butler M.G. (2014). Chromosomal Microarray Analysis of Consecutive Individuals with Autism Spectrum Disorders or Learning Disability Presenting for Genetic Services. Gene.

[17] Yin K.-J., Hamblin M., Fan Y., Zhang J., Chen Y.E. (2015). Krüpple-like Factors in the Central Nervous System: Novel Mediators in Stroke. Metab. Brain Dis..

[18] Ray S.K. (2016). The Transcription Regulator Kruppel-Like Factor 4 and Its Dual Roles of Oncogene in Glioblastoma and Tumor Suppressor in Neuroblastoma. Immunopathol. Dis. Ther..

[19] Spielmann M., Reichelt G., Hertzberg C., Trimborn M., Mundlos S., Horn D., Klopocki E. (2011). Homozygous Deletion of Chromosome 15q13.3 Including CHRNA7 Causes Severe Mental Retardation, Seizures, Muscular Hypotonia, and the Loss of KLF13 and TRPM1 Potentially Cause Macrocytosis and Congenital Retinal Dysfunction in Siblings. Eur. J. Med. Genet..

[20] Zhang S., Zhang X., Purmann C., Ma S., Shrestha A., Davis K.N., Ho M., Huang Y., Pattni R., Wong W.H. (2021). Network Effects of the 15q13.3 Microdeletion on the Transcriptome and Epigenome in Human-Induced Neurons. Biol. Psychiatry.

[21] Körner M.B., Velluva A., Bundalian L., Radtke M., Lin C.-C., Zacher P., Bartolomaeus T., Kirstein A.S., Mrestani A., Scholz N. (2022). Altered Gene Expression Profiles Impair the Nervous System Development in Individuals with 15q13.3 Microdeletion. Sci. Rep..

[22] Malwade S., Gasthaus J., Bellardita C., Andelic M., Moric B., Korshunova I., Kiehn O., Vasistha N.A., Khodosevich K. (2022). Identification of Vulnerable Interneuron Subtypes in 15q13.3 Microdeletion Syndrome Using Single-Cell Transcriptomics. Biol. Psychiatry.

[23] Wiemerslage L., Islam R., van der Kamp C., Cao H., Olivo G., Ence-Eriksson F., Castillo S., Larsen A.L., Bandstein M., Dahlberg L.S. (2017). A DNA Methylation Site within the KLF13 Gene Is Associated with Orexigenic Processes Based on Neural Responses and Ghrelin Levels. Int. J. Obes..

[24] Ávila-Mendoza J., Subramani A., Sifuentes C.J., Denver R.J. (2020). Molecular Mechanisms for Krüppel-Like Factor 13 Actions in Hippocampal Neurons. Mol. Neurobiol..

[25] Bernhardt C., Sock E., Fröb F., Hillgärtner S., Nemer M., Wegner M. (2022). KLF9 and KLF13 Transcription Factors Boost Myelin Gene Expression in Oligodendrocytes as Partners of SOX10 and MYRF. Nucleic Acids Res..

[26] Lavallée G., Andelfinger G., Nadeau M., Lefebvre C., Nemer G., Horb M.E., Nemer M. (2006). The Kruppel-like Transcription Factor KLF13 Is a Novel Regulator of Heart Development. EMBO J..

[27] Li W., Li B., Li T., Zhang E., Wang Q., Chen S., Sun K. (2020). Identification and Analysis of KLF13 Variants in Patients with Congenital Heart Disease. BMC Med. Genet..

[28] Wang S.-S., Wang T.-M., Qiao X.-H., Huang R.-T., Xue S., Dong B.-B., Xu Y.-J., Liu X.-Y., Yang Y.-Q. (2020). KLF13 Loss-of-Function Variation Contributes to Familial Congenital Heart Defects. Eur. Rev. Med. Pharm. Sci..

[29] Zeng N., Jian Z., Zhu W., Xu J., Fan Y., Xiao F. (2023). KLF13 Overexpression Protects Sepsis-induced Myocardial Injury and LPS-induced Inflammation and Apoptosis. Int. J. Exp. Pathol..

[30] Henson B.J., Gollin S.M. (2010). Overexpression of *KLF13* and *FGFR3* in Oral Cancer Cells. Cytogenet. Genome Res..

[31] Wang Q., Peng R., Wang B., Wang J., Yu W., Liu Y., Shi G. (2018). Transcription Factor KLF13 Inhibits AKT Activation and Suppresses the Growth of Prostate Carcinoma Cells. Cancer Biomark..

[32] Li B., Pang S., Dou J., Zhou C., Shen B., Zhou Y. (2022). The Inhibitory Effect of LINC00261 Upregulation on the Pancreatic Cancer EMT Process Is Mediated by KLF13 via the MTOR Signaling Pathway. Clin. Transl. Oncol..

[33] Ding Y., Xu Y., Fu Y., Zhang H., Zhao L., Fan X. (2022). Kruppel-like Factor 13 Inhibits Cell Proliferation of Gastric Cancer by Inducing Autophagic Degradation of β-Catenin. Discov. Oncol..

[34] Khan K., Gulzar A., Badshah Y., Ashraf N.M., Raq M., Hamid A., Shabbir M., Afsar T., Almajwal A., Arshad M. (2022). Determining KLF13 Structure, Uniqueness, and Possible Molecular Crosstalk in Prostate Cancer.

[35] Vinci M., Costanza C., Galati Rando R., Treccarichi S., Saccone S., Carotenuto M., Roccella M., Calì F., Elia M., Vetri L. (2023). STXBP6 Gene Mutation: A New Form of SNAREopathy Leads to Developmental Epileptic Encephalopathy. Int. J. Mol. Sci..

[36] Calì F., Forster P., Kersting C., Mirisola M.G., D’Anna R., De Leo G., Romano V. (2002). DXYS156: A Multi-Purpose Short Tandem Repeat Locus for Determination of Sex, Paternal and Maternal Geographic Origins and DNA Fingerprinting. Int. J. Leg. Med..

[37] Desvignes J.-P., Bartoli M., Delague V., Krahn M., Miltgen M., Béroud C., Salgado D. (2018). VarAFT: A Variant Annotation and Filtration System for Human next Generation Sequencing Data. Nucleic Acids Res..

[38] Richards S., Aziz N., Bale S., Bick D., Das S., Gastier-Foster J., Grody W.W., Hegde M., Lyon E., Spector E. (2015). Standards and Guidelines for the Interpretation of Sequence Variants: A Joint Consensus Recommendation of the American College of Medical Genetics and Genomics and the Association for Molecular Pathology. Genet. Med..

[39] Kopanos C., Tsiolkas V., Kouris A., Chapple C.E., Albarca Aguilera M., Meyer R., Massouras A. (2019). VarSome: The Human Genomic Variant Search Engine. Bioinformatics.

[40] Quinodoz M., Royer-Bertrand B., Cisarova K., Di Gioia S.A., Superti-Furga A., Rivolta C. (2017). DOMINO: Using Machine Learning to Predict Genes Associated with Dominant Disorders. Am. J. Hum. Genet..

[41] Treccarichi S., Calì F., Vinci M., Ragalmuto A., Musumeci A., Federico C., Costanza C., Bottitta M., Greco D., Saccone S. (2024). Implications of a De Novo Variant in the SOX12 Gene in a Patient with Generalized Epilepsy, Intellectual Disability, and Childhood Emotional Behavioral Disorders. Curr. Issues Mol. Biol..

[42] Tessarech M., Friocourt G., Marguet F., Lecointre M., Le Mao M., Díaz R.M., Mignot C., Keren B., Héron B., De Bie C. (2024). De Novo Variants in SP9 Cause a Novel Form of Interneuronopathy Characterized by Intellectual Disability, Autism Spectrum Disorder, and Epilepsy with Variable Expressivity. Genet. Med..

[43] Ávila-Mendoza J., Delgado-Rueda K., Urban-Sosa V.A., Carranza M., Luna M., Martínez-Moreno C.G., Arámburo C. (2023). KLF13 Regulates the Activity of the GH-Induced JAK/STAT Signaling by Targeting Genes Involved in the Pathway. Int. J. Mol. Sci..

[44] Koh I.-U., Lee H.-J., Hwang J.-Y., Choi N.-H., Lee S. (2017). Obesity-Related CpG Methylation (Cg07814318) of Kruppel-like Factor-13 (KLF13) Gene with Childhood Obesity and Its Cis-Methylation Quantitative Loci. Sci. Rep..

[45] Kim C.K., He P., Bialkowska A.B., Yang V.W. (2017). SP and KLF transcription factors in digestive physiology and diseases. Gastroenterology.

[46] Enomoto T., Ohashi K., Shibata R., Kambara T., Uemura Y., Yuasa D., Kataoka Y., Miyabe M., Matsuo K., Joki Y. (2013). Transcriptional Regulation of an Insulin-Sensitizing Adipokine Adipolin/CTRP12 in Adipocytes by Krüppel-Like Factor 15. PLoS ONE.

[47] Jiang S., Wei H., Song T., Yang Y., Zhang F., Zhou Y., Peng J., Jiang S. (2015). KLF13 Promotes Porcine Adipocyte Differentiation through PPARγ Activation. Cell Biosci..

[48] Scohy S., Gabant P., Van Reeth T., Hertveldt V., Drèze P.-L., Van Vooren P., Rivière M., Szpirer J., Szpirer C. (2000). Identification of KLF13 and KLF14 (SP6), Novel Members of the SP/XKLF Transcription Factor Family. Genomics.

[49] Swamynathan S.K. (2010). Krüppel-like Factors: Three Fingers in Control. Hum. Genom..

[50] Pearson R.C.M., Funnell A.P.W., Crossley M. (2011). The Mammalian Zinc Finger Transcription Factor Krüppel-like Factor 3 (KLF3/BKLF). IUBMB Life.

[51] Camacho-Vanegas O., Till J., Miranda-Lorenzo I., Ozturk B., Camacho S.C., Martignetti J.A. (2013). Shaking the Family Tree: Identification of Novel and Biologically Active Alternatively Spliced Isoforms across the KLF Family of Transcription Factors. FASEB J..

[52] Quach T.T., Stratton H.J., Khanna R., Kolattukudy P.E., Honnorat J., Meyer K., Duchemin A.-M. (2021). Intellectual Disability: Dendritic Anomalies and Emerging Genetic Perspectives. Acta Neuropathol..

[53] Alemany-González M., Gener T., Nebot P., Vilademunt M., Dierssen M., Puig M.V. (2020). Prefrontal–Hippocampal Functional Connectivity Encodes Recognition Memory and Is Impaired in Intellectual Disability. Proc. Natl. Acad. Sci. USA.

[54] Xian W., Cao J., Yuan X., Wang G., Jin Q., Zhang H., Zhou G., You L. (2021). Deficiency of Intellectual Disability-Related Gene Brpf1 Attenuated Hippocampal Excitatory Synaptic Transmission and Impaired Spatial Learning and Memory Ability. Front. Cell Dev. Biol..

[55] Zhang W., Hong S., Maniar K.P., Cheng S., Jie C., Rademaker A.W., Krensky A.M., Clayberger C. (2016). KLF13 Regulates the Differentiation-Dependent Human Papillomavirus Life Cycle in Keratinocytes through STAT5 and IL-8. Oncogene.

[56] Morgan E.L., Macdonald A. (2020). Manipulation of JAK/STAT Signalling by High-Risk HPVs: Potential Therapeutic Targets for HPV-Associated Malignancies. Viruses.

[57] Martínez-Moreno C.G., Ticante-Carrizales A.G.P., González-Gallardo A., Carranza M., Luna M., Arámburo C., Ávila-Mendoza J. (2024). Designing Strategies To Enhance Gh-Dependent Axon Regeneration In Klf13 Deficient Retinal Ganglion Cells. Investig. Ophthalmol. Vis. Sci..

[58] Nicolas C.S., Peineau S., Amici M., Csaba Z., Fafouri A., Javalet C., Collett V.J., Hildebrandt L., Seaton G., Choi S.-L. (2012). The JAK/STAT Pathway Is Involved in Synaptic Plasticity. Neuron.

[59] Wang T., Yuan W., Liu Y., Zhang Y., Wang Z., Zhou X., Ning G., Zhang L., Yao L., Feng S. (2015). The Role of the JAK-STAT Pathway in Neural Stem Cells, Neural Progenitor Cells and Reactive Astrocytes after Spinal Cord Injury. Biomed. Rep..

[60] Nicolas C.S., Amici M., Bortolotto Z.A., Doherty A., Csaba Z., Fafouri A., Dournaud P., Gressens P., Collingridge G.L., Peineau S. (2013). The Role of JAK-STAT Signaling within the CNS. Jak-Stat.

[61] Qin H., Buckley J.A., Li X., Liu Y., Fox T.H., Meares G.P., Yu H., Yan Z., Harms A.S., Li Y. (2016). Inhibition of the JAK/STAT Pathway Protects Against α-Synuclein-Induced Neuroinflammation and Dopaminergic Neurodegeneration. J. Neurosci..

[62] Lashgari N.-A., Roudsari N.M., Momtaz S., Sathyapalan T., Abdolghaffari A.H., Sahebkar A. (2021). The Involvement of JAK/STAT Signaling Pathway in the Treatment of Parkinson’s Disease. J. Neuroimmunol..

[63] Jain M., Singh M.K., Shyam H., Mishra A., Kumar S., Kumar A., Kushwaha J. (2021). Role of JAK/STAT in the Neuroinflammation and Its Association with Neurological Disorders. Ann. Neurosci..

[64] Tsai S.-J. (2006). Signal Transducer and Activator of Transcription 6 (STAT6) and Attention-Deficit Hyperactivity Disorder: A Speculative Hypothesis. Med. Hypotheses.

[65] Shariq A.S., Brietzke E., Rosenblat J.D., Pan Z., Rong C., Ragguett R.-M., Park C., McIntyre R.S. (2018). Therapeutic Potential of JAK/STAT Pathway Modulation in Mood Disorders. Rev. Neurosci..

[66] Wu R., Yun Q., Zhang J., Bao J. (2019). Downregulation of KLF13 through DNMT1-Mediated Hypermethylation Promotes Glioma Cell Proliferation and Invasion. Onco Targets.

[67] Hu Y., Zhang M., Tian N., Li D., Wu F., Hu P., Wang Z., Wang L., Hao W., Kang J. (2019). The Antibiotic Clofoctol Suppresses Glioma Stem Cell Proliferation by Activating KLF13. J. Clin. Investig..

[68] Ávila-Mendoza J., Subramani A., Denver R.J. (2020). Krüppel-Like Factors 9 and 13 Block Axon Growth by Transcriptional Repression of Key Components of the CAMP Signaling Pathway. Front. Mol. Neurosci..

[69] Willcutt E.G., Nigg J.T., Pennington B.F., Solanto M.V., Rohde L.A., Tannock R., Loo S.K., Carlson C.L., McBurnett K., Lahey B.B. (2012). Validity of DSM-IV Attention Deficit/Hyperactivity Disorder Symptom Dimensions and Subtypes. J. Abnorm. Psychol..

[70] Al-Mubarak B.R., Omar A., Baz B., Al-Abdulaziz B., Magrashi A.I., Al-Yemni E., Jabaan A., Monies D., Abouelhoda M., Abebe D. (2020). Whole Exome Sequencing in ADHD Trios from Single and Multi-Incident Families Implicates New Candidate Genes and Highlights Polygenic Transmission. Eur. J. Hum. Genet..

[71] Brikell I., Kuja-Halkola R., Larsson H. (2015). Heritability of Attention-deficit Hyperactivity Disorder in Adults. Am. J. Med. Genet. Part. B Neuropsychiatr. Genet..

[72] Faraone S.V., Larsson H. (2019). Genetics of Attention Deficit Hyperactivity Disorder. Mol. Psychiatry.

[73] Arpawong T.E., Klopack E.T., Kim J.K., Crimmins E.M. (2023). ADHD Genetic Burden Associates with Older Epigenetic Age: Mediating Roles of Education, Behavioral and Sociodemographic Factors among Older Adults. Clin. Epigenetics.

[74] Kleefstra T., Kramer J.M., Neveling K., Willemsen M.H., Koemans T.S., Vissers L.E.L.M., Wissink-Lindhout W., Fenckova M., van den Akker W.M.R., Kasri N.N. (2012). Disruption of an EHMT1-Associated Chromatin-Modification Module Causes Intellectual Disability. Am. J. Hum. Genet..

[75] Vogel-Ciernia A., Wood M.A. (2014). Neuron-Specific Chromatin Remodeling: A Missing Link in Epigenetic Mechanisms Underlying Synaptic Plasticity, Memory, and Intellectual Disability Disorders. Neuropharmacology.

[76] Larizza L., Finelli P. (2019). Developmental Disorders with Intellectual Disability Driven by Chromatin Dysregulation: Clinical Overlaps and Molecular Mechanisms. Clin. Genet..

[77] Song C.-Z., Keller K., Chen Y., Stamatoyannopoulos G. (2003). Functional Interplay between CBP and PCAF in Acetylation and Regulation of Transcription Factor KLF13 Activity. J. Mol. Biol..

[78] Ahn Y.-T., Huang B., McPherson L., Clayberger C., Krensky A.M. (2007). Dynamic Interplay of Transcriptional Machinery and Chromatin Regulates “Late” Expression of the Chemokine RANTES in T Lymphocytes. Mol. Cell. Biol..

[79] Krensky A.M., Ahn Y.-T. (2007). Mechanisms of Disease: Regulation of RANTES (CCL5) in Renal Disease. Nat. Clin. Pract. Nephrol..

[80] Walton E., Pingault J.-B., Cecil C.A.M., Gaunt T.R., Relton C.L., Mill J., Barker E.D. (2017). Epigenetic Profiling of ADHD Symptoms Trajectories: A Prospective, Methylome-Wide Study. Mol. Psychiatry.

[81] Hamza M., Halayem S., Bourgou S., Daoud M., Charfi F., Belhadj A. (2019). Epigenetics and ADHD: Toward an Integrative Approach of the Disorder Pathogenesis. J. Atten. Disord..

[82] Song C.-Z., Keller K., Murata K., Asano H., Stamatoyannopoulos G. (2002). Functional Interaction between Coactivators CBP/P300, PCAF, and Transcription Factor FKLF2. J. Biol. Chem..

[83] Huang B., Ahn Y.-T., McPherson L., Clayberger C., Krensky A.M. (2007). Interaction of PRP4 with Krüppel-Like Factor 13 Regulates CCL5 Transcription. J. Immunol..

[84] Huttlin E.L., Bruckner R.J., Navarrete-Perea J., Cannon J.R., Baltier K., Gebreab F., Gygi M.P., Thornock A., Zarraga G., Tam S. (2021). Dual Proteome-Scale Networks Reveal Cell-Specific Remodeling of the Human Interactome. Cell.

[85] Bannwarth S., Ait-El-Mkadem S., Chaussenot A., Genin E.C., Lacas-Gervais S., Fragaki K., Berg-Alonso L., Kageyama Y., Serre V., Moore D.G. (2014). A Mitochondrial Origin for Frontotemporal Dementia and Amyotrophic Lateral Sclerosis through CHCHD10 Involvement. Brain.

[86] Stilling R.M., Rönicke R., Benito E., Urbanke H., Capece V., Burkhardt S., Bahari-Javan S., Barth J., Sananbenesi F., Schütz A.L. (2014). K-Lysine Acetyltransferase 2a Regulates a Hippocampal Gene Expression Network Linked to Memory Formation. EMBO J..

[87] Panahzadeh F., Mirnasuri R., Rahmati M. (2020). The Effect of Endurance Training on the Expression of PRDX6 and KAT2B Genes in Hippocampus of β Amyloid-Induced Rat Model of Alzheimer’s Disease: An Experimental Study. J. Rafsanjan Univ. Med. Sci..

[88] Smigiel K.S., Parks W.C. (2017). Matrix Metalloproteinases and Leukocyte Activation. Progress in Molecular Biology and Translational Science.

[89] Beroun A., Mitra S., Michaluk P., Pijet B., Stefaniuk M., Kaczmarek L. (2019). MMPs in Learning and Memory and Neuropsychiatric Disorders. Cell. Mol. Life Sci..

