# Supplementary material for: Bioinformatic Evaluation of KLF13 Genetic Variant: Implications for Neurodevelopmental and Psychiatric Symptoms
Source: Genes (Basel). 2024 Aug 11;15(8):1056. doi: 10.3390/genes15081056 (PMC11354057; doi:10.3390/genes15081056)
Supplement: Supplementary file 1 [file genes-15-01056-s001.zip › genes-3137633-supplementary.pdf]

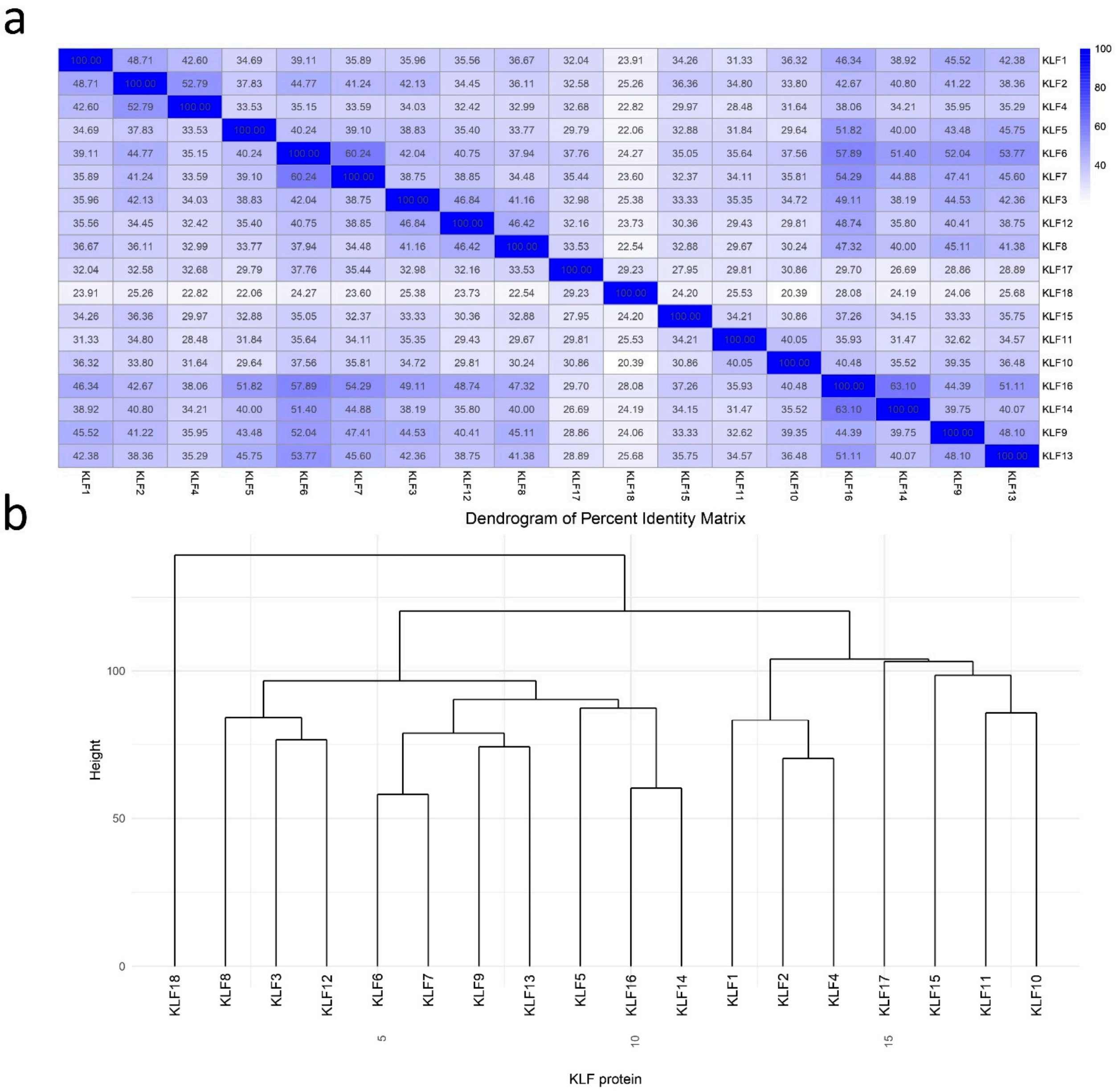


**Figure S1.** Identity level of 18 KLF proteins. (a) Heatmap related to the percentage of identity calculated for the 18 KLF proteins. The percentages were obtained from the Uniprot database using the alignment tool. (b) Dendrogram generated considering the percentage of identity among the 18 KLF proteins aligned. Both the plots were generated by R studio version 3.6.3.

**Table S1**. Variation in the hydrogen bond patterns was predicted for the wild-type and mutated KLF13 protein within the segment spanning from Alanine at position 3 to Serine at position 19.

| aa Residue | HyB aa Partner [(a)] | KLF13 (3aa-19aa) [wt] (Val7) [(b)] | KLF (3aa-19aa) [mut] (Gly7) [(c)] |
|---|---|---|---|
| Ala5 | His9 | 0 | 1 |
| Gly7[mut] | Ala11 | - | 1 |
| Asp8 | Ala12 | 0 | 1 |
| His9 | Ala5 | 0 | 1 |
| | Glu13 | 0 | 1 |
| Phe10 | Tyr6 | 0 | 1 |
| | Cys14 | 0 | 2 |
| Ala11 | Gly7[mut] | - | 1 |
| | Leu15 | 0 | 1 |
| Ala12 | Asp8 | 0 | 1 |
| | Val16 | 0 | 1 |
| Glu13 | His9 | 0 | 1 |
| | Se17 | 0 | 1 |
| Cys14 | Phe10 | 0 | 2 |
| | Glu13 | 1 | 0 |
| | Met18 | 0 | 1 |
| Leu15 | Ala11 | 0 | 1 |
| | Ser19 | 0 | 2 |
| Val16 | Ala12 | 0 | 1 |
| | Ser19 | 0 | 1 |
| | Ser20 | 0 | 2 |
| Ser17 | Glu13 | 0 | 1 |
| | Ser20 | 0 | 1 |
| Met18 | Cys14 | 0 | 1 |
| Ser19 | Leu15 | 0 | 2 |
| | Val16 | 0 | 1 |
| | Ser20 | 1 | 0 |

[(a)] Amino acid (aa) involved in hydrogen bond (HyB) with the aa residue enumerated in the first column; [(b)] number of hydrogen bonds in the wild-type KLF13 protein; [(c)] number of hydrogen bonds in the mutated KLF13 protein.
